# Polypharmacy and Drug-Drug Interactions in Elderly Patients With Gastrointestinal Bleeding: A Single-Center Retrospective Study

**DOI:** 10.7759/cureus.77866

**Published:** 2025-01-23

**Authors:** Tugce Uskur, Bedriye Feyza Kurt

**Affiliations:** 1 Medical Pharmacology, Kırklareli University Faculty of Medicine, Kırklareli, TUR; 2 Emergency Medicine, Kırklareli Training and Research Hospital, Kırklareli, TUR

**Keywords:** drug-drug interactions, elderly patients, emergency department, gi bleeding, polypharmacy

## Abstract

Introduction: Gastrointestinal (GI) bleeding is a significant clinical presentation in elderly patients, where comorbidities, polypharmacy, and drug-drug interactions markedly increase the risk of bleeding. The aim of this study was to evaluate the types of medications prescribed during the six months prior to the patients' admission and the potential risks associated with drug interactions in patients aged 65 and older diagnosed with GI bleeding.

Methods: This retrospective study included 49 patients aged 65 and older who were admitted to the emergency department of Kırklareli Training and Research Hospital with a diagnosis of GI bleeding between January 1 and December 31, 2022. Patient demographics, type of GI bleeding (upper or lower), duration of hospital stay, clinical outcomes, and medications prescribed during the six months prior to hospitalization were recorded. Statistical analyses were performed using GraphPad Prism 8.0. Continuous variables were reported as mean±standard deviation and categorical variables were analyzed using Fisher’s exact test and Chi-square test, with a significance level of p<0.05.

Results: The study included 49 patients, comprising 25 (51.02%) males and 24 (49.98%) females, with a mean age of 78.4±7.6 years. Female patients had a significantly higher mean age than males (p=0.045). Upper GI bleeding (81.6%) was more prevalent than lower GI bleeding (18.4%), with no statistically significant gender difference (p=0.7252). The mortality rate was 10.2%, with all deceased patients being female and diagnosed with upper GI bleeding (p=0.0226). A total of 110 medications were prescribed during the six months prior to hospitalization. Drug interactions were identified in 28 patients, with 67.9% classified as moderate and 28.6% as major. NSAIDs, anticoagulants, and antidepressants were the most frequently involved drug groups, significantly heightening the risk of GI bleeding.

Conclusion: Polypharmacy and drug-drug interactions are critical factors that contribute to the increased risk of GI bleeding in elderly patients. Comprehensive evaluation of medication regimens and strategies to mitigate polypharmacy are essential for improving patient safety and outcomes in this population.

## Introduction

Gastrointestinal (GI) bleeding is a common cause of emergency department (ED) visits, with approximately 800,000 annual cases reported in the United States alone, nearly half of which require hospitalization [1]. GI bleeding, which can occur anywhere in the digestive tract from the upper esophagus to the rectum, presents with symptoms such as hematemesis (vomiting blood), melena (black, tarry stools), and hematochezia (fresh blood in stools) in patients admitted to the ED [2].

Predisposing factors for GI bleeding include being over 65 years of age, male gender, comorbid conditions, and medication use associated with these conditions. The overall mortality rate among patients presenting with GI bleeding is reported to be 7%-8.2%, with a significant proportion (19%-28%) requiring admission to the intensive care unit (ICU) for monitoring and treatment [3].

One of the leading causes of GI bleeding in elderly patients, a condition associated with significant morbidity and mortality, is medication use, particularly nonsteroidal anti-inflammatory drugs (NSAIDs). NSAIDs, including low-dose aspirin, are commonly associated with an increased risk of GI ulceration [4,5]. NSAID-induced injury results from both local effects and systemic prostaglandin inhibition. NSAID use in the elderly is closely linked to the rising prevalence of chronic pain with aging. Medication use for pain management is significantly higher among individuals aged 65 and older compared to younger populations, as pain is one of the most common reasons for seeking medical care in this age group [6,7]. NSAID-related side effects are significantly more pronounced in elderly patients with chronic pain compared to younger individuals [8]. Gastrointestinal bleeding and peptic ulcer disease occur in approximately 1% of patients receiving NSAID therapy for 3-6 months and in 2-4% of those treated for one year.

Another critical concern in elderly patients is the increased bleeding risk associated with drug-drug interactions (DDIs) due to polypharmacy. The importance of drug interactions grows with advancing age. In patients using NSAIDs, concurrent use of systemic steroids, anticoagulants, antiplatelet agents, and/or antidepressants significantly increases the risk of GI side effects [9,10]. For these reasons, NSAIDs should generally be preferred for nociceptive pain, used at the lowest effective doses, and for no longer than two weeks [11,12].

One of the key risks in elderly patients is the bleeding potential associated with oral anticoagulants, particularly warfarin. The annual risk of major bleeding in patients using warfarin ranges from 0.5% to 7.0%. Numerous drugs, including paracetamol, amiodarone, erythromycin, fluconazole, fluoxetine, metronidazole, salicylates, sulfamethoxazole, tamoxifen, and thyroid hormones, are well-documented to enhance the anticoagulant effect of warfarin [13,14]. Additionally, the medications most frequently and continuously used in the elderly population-such as antihypertensives, antihyperlipidemics, antidepressants, antiplatelets, antidiabetics, NSAIDs, antacids, antipsychotics, and antiepileptics-have the potential to interact with one another and with certain foods and beverages.

This study aims to investigate the types of medications prescribed within the last six months prior to the patients' admission to the emergency department, the drug-drug interactions associated with these medications, and the potential risks of these interactions in patients aged 65 and older who were admitted to the emergency department with a diagnosis of gastrointestinal bleeding.

## Materials and methods

Data source

This retrospective study utilized medical records of 49 patients diagnosed with GI bleeding who were admitted to Kirklareli Training and Research Hospital between January 1, 2022, and December 31, 2022. The collected data included the patients' gender, age, type of GI bleeding (upper or lower), duration of hospital stay, clinical outcomes, and details of medications prescribed during the six months prior to the patients admission. The inclusion criteria were: (1) a confirmed diagnosis of GI bleeding (in patients with suspected upper GI bleeding, endoscopic evaluation was performed to identify the bleeding source) and (2) age 65 years or older. 

Ethical considerations

As a retrospective study without direct patient interaction, informed consent was not required. The study was conducted in accordance with the Declaration of Helsinki and received approval from the Ethics Committee of Kırklareli University (30/01/2024, Number: P202300044R01-09).

Statistical analysis

All statistical analyses were performed using GraphPad Prism 8.0 software. Continuous variables are presented as mean±standard deviation (SD). The normality of data distribution was assessed using the Shapiro-Wilk test, and differences between normally distributed variables were analyzed using the Student's t-test. Comparisons of categorical variables, such as gender and bleeding types (UGIB and LGIB), were conducted using the Fisher’s exact test. Relationships between hospital stay durations and clinical outcomes (discharge, death, transfer) were evaluated using the Chi-square test.

Blood parameters (e.g., RBC, hematocrit, INR) were reported using descriptive statistics only, with no further statistical analyses performed. Additionally, prescription frequencies were analyzed descriptively, and the most frequently prescribed medications were expressed as percentages. Drug-drug interactions were categorized based on severity (minor, moderate, severe) and presented as frequencies in tables and figures. A significance level of p<0.05 was considered for all analyses.

## Results

The mean age of the patients was 78.44±7.62 years. Female patients were significantly older than male patients. Among the patients, 40 (81.6%) were diagnosed with upper gastrointestinal bleeding (UGIB), while 9 (18.4%) were diagnosed with lower gastrointestinal bleeding (LGIB). There was no statistically significant difference in the distribution of UGIB and LGIB between males and females (Table 1).

**Table 1:** General characteristics of the patients included in the study. *A t-test was used to compare the mean age between female and male patients, *Fisher’s exact test was used to evaluate the distribution of UGIB and LGIB;* Chi-square test was used to assess hospital stay durations and clinical outcomes. Min-Max: minimum-maximum; SD: standard deviation; GI: gastrointestinal; UGIB: upper gastrointestinal bleeding; LGIB: lower gastrointestinal bleeding.

Variables	Female (n=24)	Male (n=25)	Total (n=49)	Test statistic	p-value
Age (Min-Max)	65-91 (82.5)	65-91 (77)	65-91 (78)	t (df): 2.060 (47)	0.045
Mean±SD	80.6±8.0	76.3±6.7	78.4±7.6		
Type of GI bleeding					
UGIB (%)	19 (79.2%)	21 (84.0%)	40 (81.6%)	Fisher's exact test	0.7252
LGIB (%)	5 (20.8%)	4 (16.0%)	9 (18.4%)		
Hospital stay duration					
1-7 days	20 (83.3%)	21 (84.0%)	41 (83.6%)	χ² (df): 2.005 (4)	0.7349
8-14 days	1 (4.2%)	1 (4.0%)	2 (4.1%)		
≥15 days	3 (12.5%)	3 (12.0%)	6 (12.3%)		
Clinical outcomes					
Discharged (%)	16 (66.7%)	24 (96.0%)	40 (81.6%)	χ² (df): 7.583 (2)	0.0226
Deceased (%)	5 (20.8%)	0 (0%)	5 (10.2%)		
Transferred (%)	3 (12.5%)	1 (4.0%)	4 (8.2%)		

Regarding hospital stay durations, 41 (83.6%) of the patients were hospitalized for 1-7 days, two (4.1%) for 8-14 days, and six (12.3%) for 15 days or longer. The distribution of hospital stay durations did not differ significantly between males and females. Clinical outcomes revealed a higher mortality rate among female patients (5, 20.8%) compared to males (0%), which was statistically significant (Table 1).

An analysis of blood parameters revealed elevated mean levels of urea (mg/dL) and the blood urea nitrogen (BUN)/creatinine ratio. Although creatinine (mg/dL) levels were generally within normal limits, 14 (28.6%)patients had values exceeding the reference range. Regarding coagulation parameters, elevated international normalized ratio (INR) levels were observed, while platelet counts (×10^3^/µL) remained within normal limits. Hematological parameters showed decreased levels of RBC (×10^6^/µL), hematocrit, and prothrombin time (s) (Table 2).

**Table 2:** Blood parameters of patients included in the study. *No statistical tests to calculate the p-values were performed for the data presented in this table.

Parameters	Min-Max	Mean±SD
RBC (×10^6^/µL)	1.46-4.98	3.33±0.78
Hematocrit (%)	13.9-42.3	28.73±6.82
Prothrombin time (PT) (s)	11.7-201	21.45±6.12
INR (international normalized ratio) (unitless)	0.88-4.32	1.78±0.61
PLT (platelet count) (×10^3^/µL)	77-552	241.02±105.98
Creatinine (mg/dL)	0.6-3.45	1.19±0.59
Urea (mg/dL)	22-287	88.36±49.68
BUN/creatinine ratio (blood urea nitrogen) (unitless)	24.24-198.39	77.90±37.30

A total of 110 medications were prescribed to the patients during the six months prior to their admission, including 69 (62.7%) prescriptions for female patients and 41 (37.3%) for male patients. The five most frequently prescribed medications were quetiapine (7, 6.4%), enoxaparin sodium (6, 5.5%), dexketoprofen (6, 5.5%), metoprolol (6, 5.5%), and warfarin (5, 4.5%) (Table 3).

**Table 3:** Distribution of medications prescribed to patients included in the study. *Drug: name of the prescribed medication. Percentages are calculated based on the total number of prescribed drugs (n=110), *n: number of patients; %: percentage. *No statistical test was applied to calculate the p-value to this table as it represents descriptive data.

Drug	Number of patients and percentage (n, %)
Quetiapine	7 (6.4%)
Enoxaparin sodium	6 (5.5%)
Dexketoprofen	6 (5.5%)
Metoprolol	6 (5.5%)
Warfarin	5 (4.5%)

**Figure 1:**
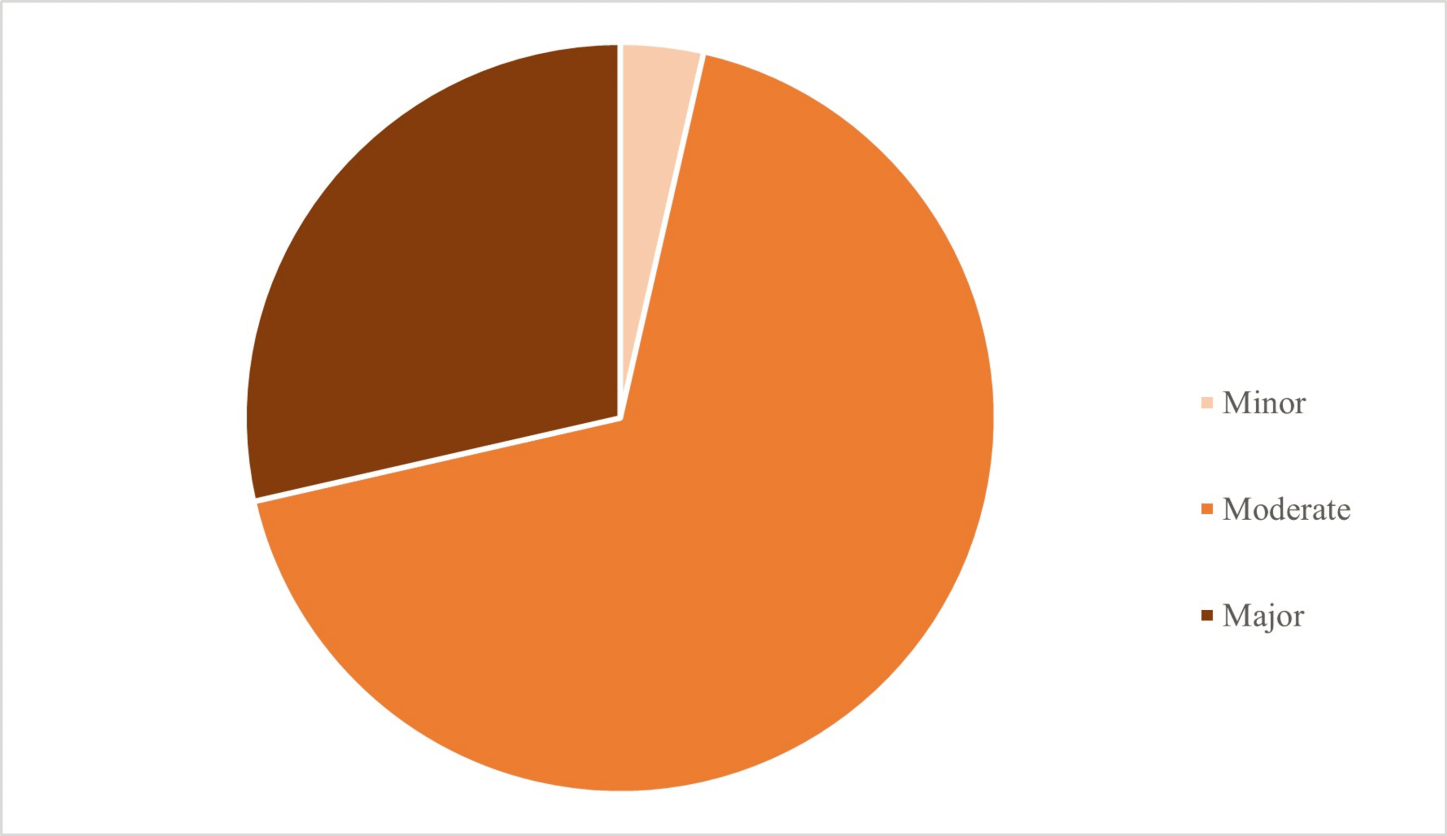
Distribution of drug-drug interaction types. The data is represented as N and % values; 1 (3.6%) minor, 19 (67.9%) moderate, and 8 (28.6%) major interactions.

When the patients' prescriptions were analyzed for drug-drug interactions, 28 (57.1%) patients were identified to have such interactions. These were classified as 1 (3.6%) minor, 19 (67.9%) moderate, and 8 (28.6%) major interactions (Figure 1). Drug interaction analyses were performed using the drugs.com database [15]. 

In this study, drug-drug interactions identified in 15 (30.6%) patients were found to pose a risk of bleeding (Table 4). The drugs involved in these interactions included five analgesics (four of which were nonsteroidal anti-inflammatory drugs (NSAIDs)), four antidepressants, three anticoagulants, three antiplatelet agents, and one antibiotic.

**Table 4:** Identified drug-drug interactions in patients included in the study. * The severity of interactions (major: severe, moderate: moderate) was obtained from drug-drug interaction databases [15].

Drug	Interacting drug	Severity	Interaction effect	Number of patients
Aspirin	Escitalopram	Major	risk of bleeding	1
Aspirin	Pentoxifylline	Moderate	risk of bleeding	1
Dexketoprofen	Escitalopram	Major	risk of bleeding	1
Dexketoprofen	Clopidogrel	Moderate	risk of bleeding	1
Dexketoprofen	Enoxaparin	Major	risk of bleeding	1
Diclofenac	Duloxetine	Moderate	risk of bleeding	1
Diclofenac	Flurbiprofen	Major	risk of bleeding	1
Enoxaparin	Escitalopram	Major	risk of bleeding	2
Fluoxetine	Apixaban	Moderate	risk of bleeding	1
Paracetamol	Warfarin	Moderate	risk of bleeding	2
Warfarin	Enoxaparin	Major	risk of bleeding	1
Warfarin	Metronidazole	Major	risk of bleeding	1
Warfarin	Sertraline	Moderate	risk of bleeding	1

## Discussion

With aging, the prevalence of comorbidities increases, leading to a higher use of medications associated with bleeding and an elevated frequency of DDIs due to polypharmacy. These factors significantly heighten the risk of GI bleeding in elderly individuals. Hospital stays, along with mortality and morbidity rates related to GI bleeding, are notably higher in older adults compared to younger populations [4]. Managing and evaluating GI bleeding in elderly patients poses substantial clinical challenges. Consequently, raising awareness about medications that may contribute to bleeding and potential DDIs in elderly patients is critically important for preventing GI bleeding. This study aimed to evaluate the medications prescribed to elderly patients presenting to the ED with a diagnosis of GI bleeding and to assess the potential risks associated with DDIs.

The gender distribution (female: 24, male: 25) and mean age (78.4±7.6 years) of the patients in our study align with the findings reported by Tomizawa et al. [16]. GI bleeding affects a substantial proportion of elderly individuals and may originate from either the upper or lower gastrointestinal tract. Approximately 70% of acute upper GI bleeding episodes occur in patients over 60 years of age [17], and the incidence increases with age [18,19]. Advanced age is also recognized as a risk factor for mortality in cases of UGIB [20]. Although less common than UGIB, the frequency of LGIB and the duration of treatment also increase with age [21]. According to Kumar and Mills [22], 70% of GI bleeding cases are of upper gastrointestinal origin. In our study, 40 (81.6%) patients were diagnosed with UGIB, while 9 (18.4%) had LGIB. All deceased patients were diagnosed with UGIB, consistent with findings in the literature.

Mortality rates among patients with GI bleeding are reported to be less than 10% in populations under 60 years but increase to 12%-25% in those over 60 years [23]. In our study, the mortality rate was 10.2%, with 5 of the 49 patients deceased. Notably, all deceased patients were female, which may be attributed to their higher average age and more frequent use of medications. However, the limited sample size in our study poses a significant limitation in interpreting mortality rates and gender differences. Future studies with larger sample sizes may provide more definitive insights.

In terms of laboratory parameters, the mean hematocrit value (28.7) in our study was below the reference range and closely aligned with the findings of Sittichanbuncha et al. [24], which reported values of 26.4 for UGIB and 32.3 for LGIB. However, the INR value (1.78) observed in our study was higher than those reported by Sittichanbuncha et al. (1.2 for UGIB and 1.1 for LGIB). This discrepancy may be attributed to the frequent use of medications that elevate INR levels among a significant proportion of the patients in our cohort. Additionally, elevated urea levels and BUN/creatinine ratios in our data further support this hypothesis. Although similar studies have reported BUN/creatinine ratios exceeding reference ranges, the values observed in our study were notably higher [24,25].

Regarding medications, a total of 110 drugs were prescribed during the six months prior to the patients' admission, with analgesics, psychiatric drugs, cardiovascular drugs, and anticoagulants being the most commonly used categories. The most frequently prescribed medications were quetiapine (n=7, 6.4%), enoxaparin sodium (n=6, 5.5%), dexketoprofen (n=6, 5.5%), metoprolol (n=6, 5.5%), and warfarin (n=5, 4.5%) (Table 3). Anticoagulants (e.g., enoxaparin sodium and warfarin) and NSAIDs (e.g., dexketoprofen) are well-documented to increase the risk of GI bleeding [4,22]. Given the high prevalence of chronic diseases in elderly patients, the frequent use of these medications likely exacerbates the risk of bleeding.

Polypharmacy and the concurrent use of specific medications were identified as major contributors to DDIs in our study. A total of 28 patients (100%) experienced DDIs, with 19 (67.9%) interactions classified as moderate and 8 (28.6%) as major (Table 4). These interactions represent significant risk factors that may adversely impact the clinical outcomes of patients. Among the 15 patients (100%) with DDIs posing a bleeding risk, analgesics were the most frequently involved (n=5, 33.3%), with four of these drugs belonging to the NSAID group. Previous studies have demonstrated that NSAIDs significantly increase the risk of GI bleeding, especially when combined with anticoagulants or antidepressants [9,22]. The concomitant use of selective serotonin reuptake inhibitors (SSRIs) with anticoagulants is also known to markedly elevate bleeding risk. For instance, a retrospective study reported that the combination of escitalopram and enoxaparin increased the risk of major bleeding by 18.85 times. This elevated risk has been associated with clinical outcomes such as epistaxis, ecchymosis, petechiae, and even life-threatening bleeding events. Additionally, strong evidence in the literature suggests that the concurrent use of NSAIDs and SSRIs increases the risk of both cranial and GI bleeding [26-28]. The interactions observed in our study are consistent with these findings.

These findings underscore the serious issue of polypharmacy in elderly patients. Interactions between commonly used drugs, such as NSAIDs, and other drug classes can result in severe clinical outcomes, including bleeding. Therefore, the careful evaluation of prescribed medications and the implementation of strategies to minimize polypharmacy is crucial for improving patient safety and clinical outcomes.

Limitations

The main limitation of this study is that only medications prescribed within the hospital system were included, while drugs obtained outside the hospital or without a prescription were not considered. Additionally, the retrospective design of the study resulted in certain data limitations. Furthermore, the relatively small sample size restricts the generalizability of the findings, potentially limiting the ability to accurately reflect the distribution of drug-drug interactions in larger populations.

## Conclusions

In conclusion, this study highlights that ageing, increased comorbidity rates, and drug-drug interactions associated with polypharmacy significantly contribute to the elevated risk of gastrointestinal bleeding in elderly individuals. The concurrent use of NSAIDs, anticoagulants, and antidepressants emerged as a primary factor driving this heightened risk. Most drug-drug interactions observed in this study were of moderate or major severity, with the potential to negatively impact clinical outcomes. To mitigate bleeding risks in elderly patients, a careful evaluation of prescribed medications, strategies to minimize polypharmacy, and a multidisciplinary approach are essential. Future prospective studies with larger sample sizes are necessary to gain deeper insights into this critical issue.
